# Exosomes Derived from Dendritic Cells Treated with *Schistosoma japonicum* Soluble Egg Antigen Attenuate DSS-Induced Colitis

**DOI:** 10.3389/fphar.2017.00651

**Published:** 2017-09-14

**Authors:** Lifu Wang, Zilong Yu, Shuo Wan, Feng Wu, Wei Chen, Beibei Zhang, Datao Lin, Jiahua Liu, Hui Xie, Xi Sun, Zhongdao Wu

**Affiliations:** ^1^Department of Parasitology of Zhongshan School of Medicine, Sun Yat-sen University Guangzhou, China; ^2^Key Laboratory of Tropical Disease Control, Ministry of Education, Sun Yat-sen University Guangzhou, China; ^3^Provincial Engineering Technology Research Center for Biological Vector Control Guangzhou, China; ^4^Department of Clinical Laboratory, The Sixth Affiliated Hospital of Sun Yat-sen University Guangzhou, China; ^5^Department of Pancreatobiliary Surgery, The First Affiliated Hospital of Sun Yat-sen University Guangzhou, China

**Keywords:** soluble egg antigen, dendritic cell, exosomes, dextran sulfate sodium, inflammatory bowel disease

## Abstract

Exosomes are 30–150 nm small membrane vesicles that are released into the extracellular medium via cells that function as a mode of intercellular communication. Dendritic cell (DC)-derived exosomes modulate immune responses and prevent the development of autoimmune diseases. Moreover, *Schistosoma japonicum* eggs show modulatory effects in a mouse model of colitis. Therefore, we hypothesized that exosomes derived from DCs treated with *S. japonicum* soluble eggs antigen (SEA; SEA-treated DC exosomes) would be useful for treating inflammatory bowel disease (IBD). Exosomes were purified from the supernatant of DCs treated or untreated with SEA and identified via transmission electron microscopy, western blotting and NanoSight. Acute colitis was induced via the administration of dextran sulfate sodium (DSS) in drinking water (5.0%, wt/vol). Treatment with exosomes was conducted via intraperitoneal injection (i.p.; 50 μg per mouse) from day 0 to day 6. Clinical scores were calculated based on weight loss, stool type, and bleeding. Colon length was measured as an indirect marker of inflammation, and colon macroscopic characteristics were determined. Body weight loss and the disease activity index of DSS-induced colitis mice decreased significantly following treatment with SEA-treated DC exosomes. Moreover, the colon lengths of SEA-treated DC exosomes treated colitis mice improved, and their mean colon macroscopic scores decreased. In addition, histologic examinations and histological scores showed that SEA-treated DC exosomes prevented colon damage in acute DSS-induced colitis mice. These results indicate that SEA-treated DC exosomes attenuate the severity of acute DSS-induced colitis mice more effectively than DC exosomes. The current work suggests that SEA-treated DC exosomes may be useful as a new approach to treat IBD.

## Introduction

Inflammatory bowel disease (IBD), including both Crohn’s disease (CD) and ulcerative colitis (UC), is characterized by the chronic and remittent-relapsing inflammation of the intestinal tract. IBD affects millions of people worldwide (Hanauer, 2006; Maloy and Powrie, 2011). Although its etiology remains debated, IBD likely results from a combination of environmental, genetic and immunoregulatory factors (Gracie and Ford, 2015). Bloody diarrhea, abdominal discomfort, fever, and weight loss are the most common symptoms of patients with IBD (Rönnblom et al., 2010; Boldeanu et al., 2014). Currently, the treatment of IBD primarily consists of 5-aminosalicylic acid (5-ASA) agents, steroids, antimicrobials, and select immunomodulators (Baumgart and Sandborn, 2007). However, these drugs have limitations, and many patients cannot achieve remission. The exploration of novel therapeutic methods is urgently needed. For exploring novel therapeutic methods of IBD, various studies have focused on the potential value of herbal extract, such as *Scutellariae radix*, *Aloysia triphylla*, *Harpagophytum procumbens* and *Chamomile*, and found that the herbal extracts have therapeutic effect in experimental model of IBD (Chung et al., 2007; Lenoir et al., 2012; Menghini et al., 2016; Locatelli et al., 2017).

Recently, the hygiene hypothesis proposed that epidemiological investigations and experimental data have shown that less exposure to parasite infections during early childhood increases susceptibility to immune disorders such as IBD (Moreels and Pelckmans, 2005; Ramanan et al., 2016). Studies have demonstrated that helminth products protect against autoimmunity via the innate Type 2 cytokines IL-5 and IL-33 (Finlay et al., 2016). Furthermore, *Schistosoma mansoni* and *Ancylostoma caninum* soluble proteins hold therapeutic potential to treat 2,4,6-trinitrobenzene sulfonic acid (TNBS)-induced colitis in mice (Ruyssers et al., 2009). *A. caninum* excretory/secretory products can ameliorate the pathogenesis of colitis in mice (Sotillo et al., 2017), and *Schistosoma japonicum* and *S. mansoni* eggs have modulatory effects on colitis in mice (Yang et al., 2007; Xia et al., 2011; Hasby et al., 2015). Therefore, parasite therapy has been suggested; however, this treatment has potentially harmful effects.

Exosomes are 30–150 nm small membrane vesicles that are released into the extracellular medium by most cells (Thery et al., 2002; Boyiadzis and Whiteside, 2017). They mediate the transfer of proteins, genes (RNA, miRNA, and DNA) and lipids both *in vitro* and *in vivo*, and they function as a mode of intercellular communication (Boyiadzis and Whiteside, 2017). Previous studies have demonstrated that exosomes are involved in immunoregulation mechanisms including modulating antigen presentation, immune activation, immune suppression, and immune surveillance (Greening et al., 2015). Because exosomes have less cytotoxicity and biohazardous potential, and because the proteins and nucleic acids that they carry are not easily degraded, interest in the clinical applications of exosomes for therapy has grown; in fact, they exhibit more advantages than parental cells (Marcus and Leonard, 2013). Such as, dendritic cell (DC)-derived exosomes modulate the immune response and prevent the development of autoimmune diseases (Yang et al., 2010; Yin et al., 2013; Greening et al., 2015).

Because *S. japonicum* eggs have modulatory effects on colitis mice, and DC-derived exosomes prevent the development of autoimmune diseases, we hypothesized that exosomes derived from DCs treated with *S. japonicum* soluble egg antigen (SEA; SEA-treated DC exosomes) would have utility in the treatment of IBD. In the current study, SEA-treated DC exosomes were isolated from the supernatant of a culture of SEA-treated immature bone marrow-derived DCs (BMDCs). We found that SEA-treated DC exosomes attenuated dextran sulfate sodium (DSS)-induced colitis.

## Materials and Methods

### Animals and Ethics

Male BALB/c mice aged 6 weeks and weighing 18–20 g were purchased from the experimental animal center of Sun Yat-sen University. All animal experimental procedures were approved by the Sun Yat-sen University Committee for Animal Research and conformed to the Guide for the Care and Use of Laboratory Animals of the National Institute of Health in China.

### DC Generation and Exosome Purification

Bone marrow-derived DCs were generated using methods described by Inaba et al. (1992) and Lutz et al. (1999). Briefly, BALB/c mice tibiae were removed and left in 70% ethanol for 2–5 min for disinfection and then washed in PBS. Cells within the marrow were disintegrated by vigorous pipetting. Thereafter, cells were cultured in medium RPMI-1640 (GIBCO, Germany) supplemented with Penicillin (100 U/ml, Sigma, Germany), Streptomycin (100 μg/ml, Sigma, Germany), L-glutamin (2 mM, Sigma, Germany), 10% heat-inactivated fetal calf serum (GIBCO, Germany), recombinant murine granulocyte–macrophage colony-stimulating factor (200 U/ml, perprotech, United States), recombinant murine interleukin-4 (5 ng/ml, perprotech, United States). At days 3, 5, 7 and 9, half of the cells culture supernatant was collected and centrifuged, the cell pellet resuspended and given back to the original plate, and then adding the equivalent of the medium. At day 10, cells can be used (BMDCs). The prepared BMDCs were treated with SEA (40 μg/ml) or untreated; after incubation for 24 h, the culture supernatant was harvested. Exosomes were purified from the supernatant using an exosome extraction kit (Invitrogen, United States) according to manufacturer’s instructions.

### Electron Microscopy and NanoSight

Negative-staining transmission electron microscopy (TEM) was conducted to analyze the exosomes. Exosomes suspended in 2% glutaraldehyde were loaded on a copper grid and negatively stained with 3% (w/v) aqueous phosphotungstic acid for 1 min. The grid was then examined using an FEI Tecnai G2 Sprit Twin transmission electron microscope.DC exosome particles were analyzed using NanoSight NS300 (Malvern Instruments, United Kingdom).

### Western Blotting

Dendritic cell exosomes were lysed in a western blotting lysis buffer, separated using 10% sodium dodecyl sulfate-polyacrylamide gel electrophoresis (SDS-PAGE), and subsequently electrotransferred onto a nitrocellulose blotting membrane (GE Healthcare Life Science, United Kingdom). The membranes were incubated with anti-mouse CD63, CD9, CD81 (BD Biosciences, United States) and Calnexin (Abcam, United Kingdom) antibodies and horseradish peroxidase (HRP) conjugated anti-mouse IgG (Jackson, United States), followed by chemiluminescent detection (Amersham, United States).

### Induction of Colitis and Treatment

Acute colitis was induced via the administration of DSS (molecular mass 36–50 kDa; MP Biomedicals, Illkirch, France) in drinking water (5.0%, wt/vol). The control group received regular drinking water. Treatments with exosomes or SEA were conducted via intraperitoneal (i.p.) injection (50 μg per mouse) from day 0 to day 6. Control groups received the same volume of vehicle (PBS). There were five mice in each group, and each group was used for three independent experiments.

### Clinical Scoring of Disease

Mice were weighed daily, and the occurrence of diarrhea and bleeding were recorded. Scores were evaluated based on weight loss, stool type, and bleeding (**Table 1**) as described by Sann et al. (2013). Briefly, weight loss was scored as 0 (< 2%), 1 (≥ 2%– < 5%), 2 (≥ 5%– < 10%), 3 (≥ 10%– < 15%) or 4 (≥ 15%); stool was scored as 0 (normal), 1 (softer stool), 2 (moderate diarrhea), or 3 (diarrhea); and bleeding was scored as 0 (no rectal bleeding), 1 (weak hemoccult), 2 (blood in stool), or 3 (fresh rectal bleeding). The summed scores for weight loss, diarrhea, and bleeding were used as the clinical disease score [disease activity index (DAI)]. All scores were assessed by an independent observer blinded to the treatment.

**Table 1:** Assessment of the DAI.

Body weight loss (%)	Stool type	Bleeding	Score
<2%	Normal	No rectal bleeding	0
≥2–<5%	Softer stool	Weak hemoccult	1
≥5–<10%	Moderate diarrhea	Visual blood in stool	2
≥10–<15%	Diarrhea	Fresh rectal bleeding	3
≥15%	–	–	4


### Macroscopic Assessment and Histologic Analysis

On day 6, the animals were sacrificed, and their colons were removed. Colon length was measured as an indirect marker of inflammation. An independent observer who was blinded to treatment status determined the macroscopic characteristics. Briefly, the macroscopic scores were 0 (no damage), 1 (hyperemia without ulcers), 2 (hyperemia and wall thickening without ulcers), 3 (one ulceration site without wall thickening), 4 (two or more ulceration sites), 5 (0.5-cm extension of inflammation or major damage), and 6–10 (1-cm extension of inflammation or severe damage; **Table 2**; Yang et al., 2016).

**Table 2:** Assessment of macroscopic scores.

Colon damage	Score
No damage	0
Hyperemia without ulcers	1
Hyperemia and wall thickening without ulcers	2
One ulceration site without wall thickening	3
Two or more ulceration sites	4
0.5 cm extent of inflammation or major damage	5
1 cm extent of inflammation or severe damage	6–10


Colons were fixed overnight in 4% (w/v) paraformaldehyde. Five-millimeter paraffin sections were stained with hematoxylin and eosin (H&E). The histopathological score was examined in a blinded fashion according to the criteria described by Sann et al. (2013). The following parameters were evaluated: extent of inflammation, infiltration of neutrophils and lympho-histiocytes, extent of crypt damage, crypt abscess, sub-mucosal edema, loss of goblet cells, and reactive epithelial hyperplasia (**Table 3**). The aggregated individual histopathological scores were calculated.

**Table 3:** Assessment of histopathological scores.

Grade	Extent of inflammation	Infiltration neutrophils+ lympho-histiocytes	Extent of crypt damage	Crypt abscesses	Sub-mucosal edema	Loss of goblet cells	Reactive epithelial hyperplasia
0	None	None	None	None	None	None	None
1	Mucosa	Focal	Basal one third	Focal	Focal	Focal	Focal
2	Mucosa+ submucosa	Multifocal	Basal two thirds	Multifocal	Multifocal	Multifocal	Multifocal
3	Mucosa+submucosa+ muscle layer	Diffuse	Entire crypt damage		Diffuse	Diffuse	Diffuse
4	Transmura		Crypt damage+ulceration				


### RNA Extraction and Real-Time PCR

RNA was extracted was by Trizol reagent (Invitrogen, United States), according to the manufacturer’s instructions. Total RNA (1000 ng) was converted to cDNA by a Thermo Scientific RevertAid First Strand cDNA Synthesis Kit (Thermo Scientific, United States). PCR reactions were performed using SYBR Green QPCR Master Mix (TaKaRa, Japan), according to manufacturer’s instructions. The reaction over 40 cycles with denaturation at 95°C for 30 s followed by 95°C for 5 s and 60°C for 20 s. The primers of tumor necrosis factor-alpha (TNF-α), interferon-gamma (IFN-γ), interleukin-17 (IL-17A), interleukin-12 (IL-12), interleukin-22 (IL-22) and transforming growth factor (TGF)-β are listed in **Table 4**.

**Table 4:** Primers used for real-time PCR analysis.

Genes	Primer	sequence (5′→3′)
TNF-α	Forward primer	CCCTCACACTCAGATCATCTTCT
	Reverse primer	GCTACGACGTGGGCTACAG
IFN-γ	Forward primer	GGAACTGGCAAAAGGATGGTGAC
	Reverse primer	GCTGGACCTGTGGGTTGTTGAC
IL-12	Forward primer	TTGAGTGCCAATTCGATGAT
	Reverse primer	TTGAGGGCTTGTTGAGATGA
IL-17A	Forward primer	GCTCCAGAAGGCCCTCAGACT
	Reverse primer	CCAGCTTTCCCTCCGCATTGA
IL-22	Forward primer	TCAGTGCTAAGGATCAGTGCT
	Reverse primer	TGATTGCTGAGTTTGGTCAGG
TGF-β	Forward primer	AACTATTGCTTCAGCTCCACAG
	Reverse primer	AGTTGGCATGGTAGCCCTTG
GAPDH	Forward primer	ACTCCACTCACGGCAAATTC
	Reverse primer	TCTCCATGGTGGTGAAGACA


### Statistical Analyses

Data were expressed as means ± SEM. Multiple groups were compared using a one-way analysis of variance (ANOVA) followed by Dunnett’s multiple comparison *post hoc* test to determine statistical significance. Statistical significance between two experimental groups was assessed using the Independent-Sample *t*-test. A value of *P <* 0.05 was considered as significant.

## Results

### Extraction and Identification of SEA-Treated and Untreated DC Exosomes

**FIGURE 1:**
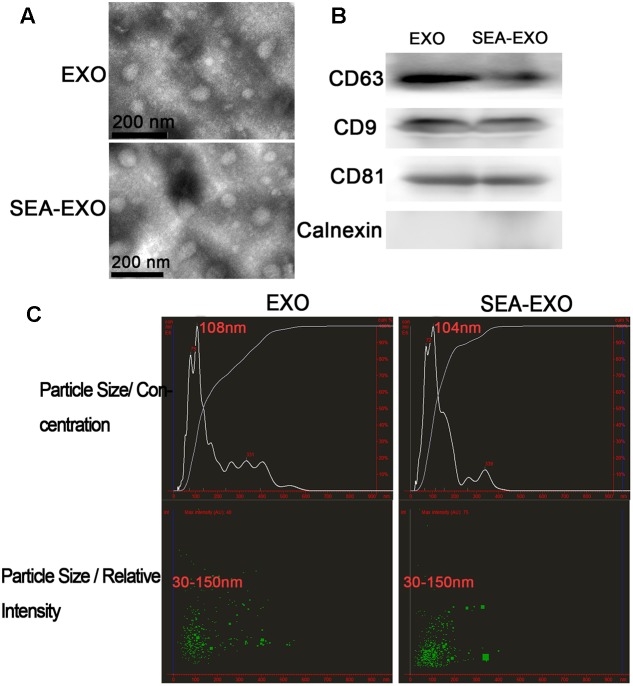
Successful isolation of exosomes from the supernatant of DCs treated with SEA (SEA-EXO) or untreated (EXO). **(A)** Exosome ultrastructure was confirmed via negative-staining TEM. **(B)** The expressions of the exosome-specific markers CD63, CD9, and CD81 were confirmed via western blotting. **(C)** The size distribution profile of the exosomes was investigated using NanoSight.

Bone marrow-derived DCs were cultured *in vitro* and either treated with SEA or left untreated. Culture supernatants were collected after 24 h. Exosomes were isolated from supernatants using exosome extraction kits according to the manufacturer’s instructions. To verify the purity and quality of the exosomes, negative-staining TEM was conducted to evaluate exosome morphology. Negative-staining TEM revealed that most untreated DC exosomes and SEA-treated DC exosomes displayed closed round vesicles with diameters of 30–150 nm (**Figure 1A**), indicating that the exosomes were undamaged during purification and uncontaminated with microparticles of cellular organelles or membranes. Furthermore, to test the expression of the exosome-specific markers, CD63, CD9, and CD81 were confirmed via western blotting; these markers were expressed at high levels (**Figure 1B**). Calnexin, the negative marker of exosomes also be determined by western blotting to test the purity of exosomes (**Figure 1B**). In addition, the size distribution profile of the exosomes was investigated using NanoSight, which revealed a size peak of 108 nm among untreated DC exosomes and 104 nm among SEA-treated DC exosomes (**Figure 1C**). These results indicate the successful isolation of the exosomes from the culture supernatants.

### SEA-Treated DC Exosomes Alleviate Clinical Scores of Acute DSS-Induced Colitis Mice

**FIGURE 2:**
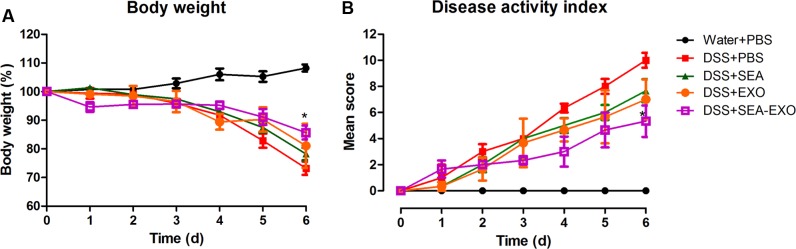
Clinical scores of acute DSS-induced colitis mice treated with exosomes. **(A)** The effect of exosomes on the body weight of mice (50 μg/mouse). Mice were weighed daily. **(B)** The effect of exosomes on the DAI. The DAI was evaluated using the parameters of weight loss, diarrhea, and bleeding. Statistical analyses were performed using a one-way ANOVA followed by Dunnett’s multiple comparison *post hoc* test (^∗^*P* < 0.05, DSS+SEA-EXO versus DSS+PBS).

To test whether SEA-treated DC exosomes attenuated colitis, acute DSS-induced colitis mice were injected i.p. with SEA-treated DC exosomes (50 μg/mouse) from day 0 to day 6. Clinical scoring was based on weight loss, diarrhea, and bleeding and expressed as the DAI. DSS treatment induced substantial weight loss in mice. After DSS administration, the mice of the DSS+PBS group lost weight over time, with a maximum body weight loss of 26.75% within 6 days (**Figure 2A**). Compared with the DSS+PBS group, weight loss in the colitis mice treated with SEA-treated DC exosomes was significantly alleviated (14.31% vs. 26.75% on day 6; *P* < 0.05). In addition, compared with untreated DC exosomes and SEA, SEA-treated DC exosomes better alleviated body weight loss (**Table 5**). Second, DSS treatment caused severe diarrhea and bleeding that persisted until the sacrifice of the colitis mice. Untreated DC exosomes, SEA-treated DC exosomes and SEA alleviated the severity of diarrhea and bleeding; however, the effect of SEA-treated DC exosomes was greater than that of DC exosomes and SEA. The DAI (the sum of the weight loss, diarrhea and bleeding scores) of the colitis mice treated with SEA-treated DC exosomes was lower than that of the colitis mice treated with PBS, SEA-untreated DC exosomes and SEA (**Figure 2B** and **Table 5**). These results suggest that SEA-treated DC exosomes attenuate clinical scores in DSS-induced colitis mice, and this effect was greater than that of SEA-untreated DC exosomes and SEA.

**Table 5:** Values of the evaluation indexes.

	Body weight loss on day 6 (mean)	DAI (mean)	Colon length (mean)	Macroscopic scores (mean)	Histopathological score (mean)	n (Three independent experiments)
Water+PBS	0.00%	0.00	10.13 cm	0.00	0	5
DSS+PBS	27.75%	10.00	5.47 cm	8.33	17	5
DSS+SEA	21.79%	7.67	7.07 cm	4.67	9.67	5
DSS+EXO	19.00%	7.00	6.30 cm	4.66	12.33	5
DSS+SEA-EXO	14.31%	5.33	8.20 cm	3.33	7.33	5


### SEA-Treated DC Exosomes Ameliorate Reduction in Colon Length and Macroscopic Scores in Acute DSS-Induced Colitis Mice

**FIGURE 3:**
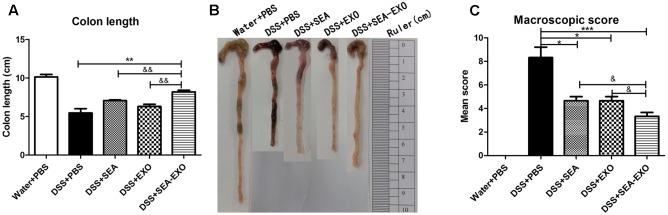
Colon length and colon macroscopic scores in acute DSS-induced colitis mice treated with exosomes. **(A)** On day 6, the mice were sacrificed, and their colons were removed. Colon length was measured as an indirect marker of inflammation. **(B)** The macroscopic appearance of colons. Mean colon length and typical injury findings are presented. **(C)** Mean macroscopic scores of colons. Statistical analyses of multiple groups were performed using a one-way ANOVA followed by Dunnett’s multiple comparison *post hoc* test versus DSS+PBS (^∗^*P <* 0.05, ^∗∗^*P <* 0.01, ^∗∗∗^*P <* 0.001). Statistical significance between two experimental groups was assessed using the Independent-Sample *t*-test (^&^*P* < 0.05, ^&&^*P* < 0.01).

Dextran sulfate sodium significantly reduces colon length in colitis mice. Therefore, the therapeutic potential of SEA-treated DC exosomes in suppressing acute colitis was investigated using colon length. The results showed that compared with the colitis mice treated with PBS, the colons of the colitis mice treated with SEA-treated DC exosomes were longer (8.2 ± 1.3 cm vs. 5.5 ± 1 cm, *P* < 0.01; **Figures 3A,B**). In addition, the colons of colitis mice treated with SEA-treated DC exosomes were longer than those in mice treated with SEA-untreated DC exosomes and SEA (**Figures 3A,B** and **Table 5**). Mean colon macroscopic scores were assessed based on hyperemia, wall thickening, ulceration, inflammation extension, and damage. Compared with the DSS+PBS group, the mean colon macroscopic scores of colitis mice treated with SEA-treated DC exosomes were significantly suppressed (**Figure 3C** and **Table 5**). In addition, the mean colon macroscopic scores of colitis mice treated with SEA-treated DC exosomes were lower than those of colitis mice treated with SEA-untreated DC exosomes and SEA (**Figure 3C** and **Table 5**). These data demonstrate that SEA-treated DC exosomes ameliorate reduction in colon length and macroscopic scores in acute DSS-induced colitis mice more effectively than DC exosomes untreated with SEA and SEA.

### SEA-Treated DC Exosomes Prevent Colon Damage in Acute DSS-Induced Colitis Mice

**FIGURE 4:**
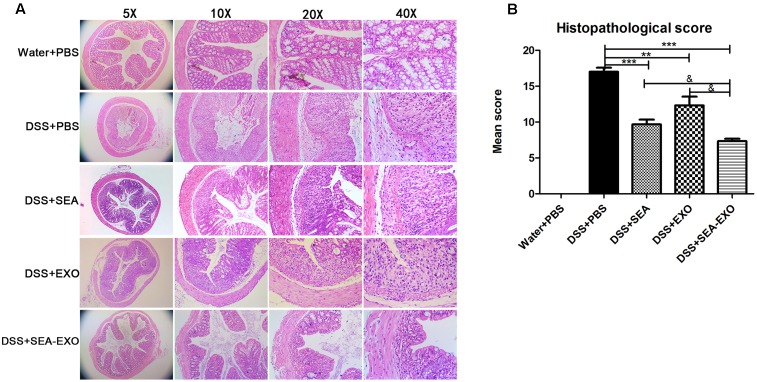
Histopathological changes in the colons of acute DSS-induced colitis mice treated with exosomes. **(A)** Colon tissue samples were examined using H&E staining (5×, 10×, 20×, and 40×). **(B)** Histopathological scores of colon tissue samples. Statistical analyses of multiple groups were performed using a one-way ANOVA followed by Dunnett’s multiple comparison *post hoc* test versus DSS+PBS (^∗∗^*P <* 0.01, ^∗∗∗^*P <* 0.001). Statistical significance between two experimental groups was assessed using the Independent-Sample *t*-test (^&^*P <* 0.05).

Finally, damage was assessed via a histologic examination. Colonic sections revealed the complete disruption of the colonic architecture in colitis mice. Colitis mice treated with SEA-treated DC exosomes retained intact colonic architecture, including reduced numbers of mucosal erosions, smaller ulcerations, lower hyperplasia, and decreased inflammatory infiltration. The damage severity in mice treated with SEA-untreated DC exosomes and SEA were greater than that in mice treated with SEA-treated DC exosomes (**Figure 4A**). Consistent with the histologic examination, the histological scores of colitis mice treated with SEA-treated DC exosomes were significantly lower than PBS-treated mice, SEA-untreated DC exosomes treated mice and SEA-treated mice (**Figure 4B** and **Table 5**). Taken together, these results indicate that SEA-treated DC exosomes prevent colon damage in acute DSS-induced colitis mice, and this effect was greater than that achieved with SEA-untreated DC exosomes and SEA.

### SEA-Treated DC Exosomes Regulate Inflammatory Cytokines Production in Acute DSS-Induced Colitis Mice

**FIGURE 5:**
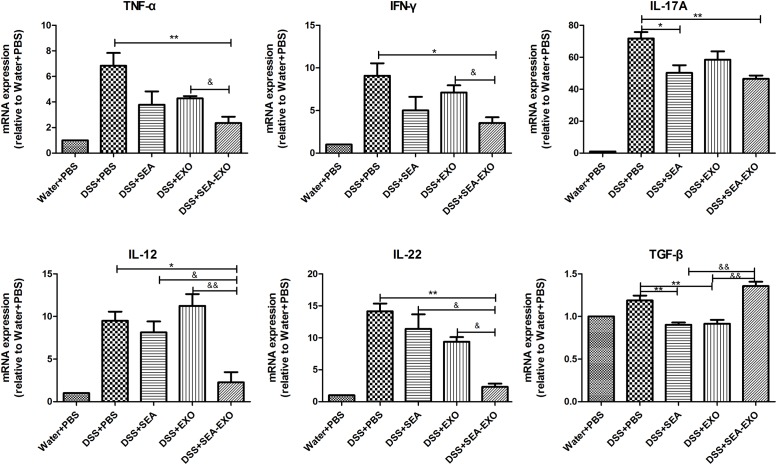
Pro-inflammatory cytokine and immunoregulatory cytokine expression in the colons of acute DSS-induced colitis were determined by Real-time PCR. The mRNA expression levels of TNF- α, IFN-γ, IL-17A, IL-12, IL-22, and TGF-β were detected by Real-time PCR, the housekeeping gene GAPDH was used as internal reference. Statistical analyses of multiple groups were performed using a one-way ANOVA followed by Dunnett’s multiple comparison *post hoc* test versus DSS+PBS (^∗^*P <* 0.05, ^∗∗^*P <* 0.01). Statistical significance between two experimental groups was assessed using the Independent-Sample *t*-test (^&^*P <* 0.05, ^&&^*P <* 0.01).

Inflammatory cytokines, such as TNF- α, IFN-γ, IL-17A, IL-12, IL-22 and TGF-β, play important roles in the pathogenesis of IBD. To determine whether SEA-treated DC exosomes can regulate the inflammatory cytokines production in colitis mice colon, the expression levels of inflammatory cytokines were examined by Real-time PCR. The results demonstrated that proinflammatory cytokines TNF- α, IFN-γ, IL-17A, IL-12, IL-22 were high expression levels in the DSS+PBS group (**Figure 5**). Compared with DSS+PBS group, TNF- α, IFN-γ, IL-17A, IL-12, IL-22 were reduced by treatment with SEA-treated DC exosomes (**Figure 5**). Furthermore, the expression levels of TNF- α, IFN-γ, IL-17A, IL-12, IL-22 in DSS+SEA-EXO group were lower than DSS+SEA group and DSS+EXO group (**Figure 5**). In addition, the expression levels of anti-inflammatory cytokines TGF-β in DSS+SEA-EXO group was higher than other groups (**Figure 5**). These results indicate that SEA-treated DC exosomes regulate inflammatory cytokines production in acute DSS-induced colitis mice.

## Discussion

Crohn’s disease and UC are the two major clinicopathological subtypes of IBD with chronic and remittent-relapsing intestinal inflammation and unclear etiology (Baumgart and Sandborn, 2012). The major factors likely responsible for IBD are environmental, genetic and immunoregulatory (De Souza and Fiocchi, 2015). Traditional treatment of IBD primarily uses 5-ASA agents, steroids, antimicrobials, and select immunomodulators (Baumgart and Sandborn, 2007). However, these drugs have limitations and adverse effects. Over the past two decades, IBD treatment via biologic therapies has demonstrated high efficacy and an excellent safety profile (Danese et al., 2014; Bonovas et al., 2016).

Exosomes are small (30–150 nm) extracellular vesicles secreted by many cells that function as key mediators and communicators between cells and transport and deliver proteins, lipids, and nucleic acids (Kourembanas, 2015). In addition, exosomes play an important role in pathophysiological processes including immune responses and inflammation, tumor growth, and infection (Barile and Vassalli, 2017). Therefore, exosomes have potential clinical applications in diagnosis, prognosis, and treatment. Evidence showed that exosomes took part in maintenance of homeostasis in intestinal mucosal immunity, including inducing oral tolerance to antigens and promoting epithelial barrier function (Xu et al., 2016). Exosomes derived from bone marrow mesenchymal stem cells can protect against TNBS-induced colitis by modulating of inflammation, suppressing oxidative stress and attenuating apoptosis (Yang et al., 2015). Exosomes released by granulocytic myeloid-derived suppressor cells attenuate DSS-induced colitis by promoting Tregs expansion and inhibiting Th1 cells proliferation (Wang et al., 2016). Furthermore, study showed that exosomes from human umbilical cord mesenchymal stem cells have effects on alleviating DSS-induced colitis through modulating IL-7 expression in macrophages (Mao et al., 2017). DCs, as the most potent professional antigen-presenting cell in the immune system, play a key role in immune responses through direct contact with other cell types and the secretion of cytokines (Romagnoli et al., 2014). DC-derived exosomes are nanometer-sized membrane vesicles secreted by DCs. Their molecular composition includes the surface expression of costimulatory molecules, functional MHC-peptide complexes and other immune function-associated molecules (Pitt et al., 2016). Previous work has shown that DC-derived exosomes can induce antitumor immunity in animal models and human clinical trials; exosomes have distinct advantages over cell-based immunotherapies involving DCs (Hao et al., 2007; Pitt et al., 2016).

Epidemiologic studies support the hypothesis that helminth parasites show great therapeutic potential for treating inflammatory diseases including IBD (Matisz et al., 2017). The protective and therapeutic effects of helminth parasite infection are corroborated by animal experiments and clinical trials. For instance, *Hymenolepis diminuta, Heligmosomoides polygyrus*, and *Trichinella spiralis* alleviate dinitrobenzene sulfonic acid-induced colitis in mice (Setiawan et al., 2007; Johnston et al., 2010; Elliott and Weinstock, 2012), *Echinococcus granulosus* has anti-inflammatory effects and protects the integrity of the intestinal mucosa in mice with DSS-induced colitis (Motomura et al., 2009), and *Ancylostoma ceylanicum* and *A. caninum* ameliorate pathology in DSS-induced colitis mice via the down-regulation of Th1 and Th17 responses (Cançado et al., 2011; Soufli et al., 2015). In addition, *S. japonicum* and *S. mansoni* eggs showed modulatory effects in a mouse model of colitis (Yang et al., 2007; Xia et al., 2011; Hasby et al., 2015).

Given that DC-derived exosomes prevent the development of autoimmune diseases and that *S. japonicum* eggs have modulatory effects on colitis in mice, we were interested in determining whether SEA-treated DC exosomes have potential uses in the treatment of IBD. The present study extracted exosomes from DC culture supernatants treated or untreated with SEA. Exosomes were identified via negative-staining TEM, NanoSight and western blotting. Exosomes displayed closed round vesicles with diameters of 30–150 nm and expressed high levels of CD63, CD9, CD81 (**Figure 1**). We subsequently treated DSS-induced colitis mice with SEA-treated DC exosomes; the results indicated that body weight loss and the DAI decreased significantly after treatment with SEA-treated DC exosomes (**Figure 2**). Moreover, after treatment with SEA-treated DC exosomes, the colon lengths of colitis mice improved, and their mean colon macroscopic scores decreased (**Figure 3**). Histologic examinations and histological scores showed that SEA-treated DC exosomes prevented colon damage in acute DSS-induced colitis mice (**Figure 4**). In addition, SEA-treated DC exosomes can regulate inflammatory cytokines production in acute DSS-induced colitis mice (**Figure 5**). These results indicate that SEA-treated DC exosomes attenuate the severity of acute DSS-induced colitis mice, and this effect is greater than that achieved using DC exosomes untreated with SEA and SEA. In conclusion, our work suggests that SEA-treated DC exosomes may be useful as a new approach to treat IBD.

## Author Contributions

LW, XS, and ZW drafted this manuscript. LW and ZY performed the experiments, analyses and interpretation of the data. SW, FW, BZ, DL, JL, and HX helped the above authors to develop the experiments. All of the authors discussed the complete dataset to establish an integral and coherent analysis. XS and ZW provided final approval of the version to be published.

## Conflict of Interest Statement

The authors declare that the research was conducted in the absence of any commercial or financial relationships that could be construed as a potential conflict of interest. The reviewer FG and handling Editor declared their shared affiliation.
